# Nonspecific Regulation of the Number of Immunocompetent Cells Under the Influence of DT Toxoid in Children With Glomerulonephritis

**DOI:** 10.3389/fimmu.2021.715389

**Published:** 2021-10-06

**Authors:** Mikhail Petrovich Kostinov, Nelli Kimovna Akhmatova, Olga Olegovna Magarshak, Anna Egorovna Vlasenko, Valentina Borisovna Polishchuk, Aristitsa Mikhailovna Kostinova, Kirill Vadimovich Mashilov

**Affiliations:** ^1^ Department of Allergology, I.I. Mechnikov Research Institute of Vaccines and Sera, Moscow, Russia; ^2^ Department of Epidemiology and Modern Vaccination Technologies, Sechenov First Moscow State Medical University, Moscow, Russia; ^3^ Department of Immunology, I.I. Mechnikov Research Institute of Vaccines and Sera, Moscow, Russia; ^4^ Department of Medical Cybernetics and Computer Science Novokuznetsk State Institute for Advanced Training of Physicians, Branch Campus of the Russian Medical Academy of Continuous Professional Education, Novokuznetsk, Russia; ^5^ Department of Immunology, NRC Institute of Immunology FMBA of Russia, Moscow, Russia

**Keywords:** glomerulonephritis, vaccination in children, post-vaccination antibodies, T-Lymphocytes, diphtheria and tetanus toxoid

## Abstract

**Background:**

Studies aimed at identifying the mechanisms of the immunoregulatory effect of vaccination with diphtheria and tetanus toxoid on the parameters of adaptive immunity in children with kidney pathology are limited. The study aimed to study the effect of revaccination against diphtheria and tetanus on the proliferation and differentiation of immunocompetent cells, the formation of specific antibodies, and the course of the disease in children with glomerulonephritis (GN).

**Methods:**

The study included 45 children with glomerulonephritis (GN) aged 5 to 15 years, in remission from 6 months up to 4 years. Of these, 25 children were revaccinated with DT toxoid (Diphtheria-Tetanus toxoid with reduced antigenic content) and 20 were in the control group (not vaccinated). The frequency of development of local and systemic reactions and the course of GN were assessed. The subpopulation structure of lymphocytes was studied in dynamics after 1-6-12 months by flow cytometry and IgG levels to diphtheria and tetanus were studied by ELISA.

**Results:**

In 92% of children with GN, the post-vaccination period was uneventful. 8% showed a rise in temperature up to 37.3^°^C, without the development of local reactions. During the year, none of the patients had an exacerbation of GN or a concomitant disease. After revaccination with DT toxoid, a significant increase in IgG antibodies against diphtheria and tetanus was revealed, which persisted after 12 months - 7.5 [5.1-10.8] IU/mL (p <0.001) and 7.2 [4.8-10.7] IU/mL (p <0.001), respectively. In the post-vaccination period, a multidirectional change in the concentration of T-lymphocytes was noted: with an initially increased level, their percentage after revaccination with DT toxoid decreases from 83 (81-86) % to 78 (76-80)% after a month (p = 0.04) and up to 75 (69-79)% after 12 months (p<0.001). In the control group, such a decrease was not observed. A similar picture was observed for T-helpers, cytotoxic T-lymphocytes, and in patients with an initially low percentage of cytotoxic T-lymphocytes, on the contrary, its increase was noted (p<0.001), which is comparable with the value of this parameter in the group of children with initially normal value (H = 0.54, p = 0.76). The same patterns were observed in the change in the content of B-cells: one month after revaccination, the relative level of B-cells in patients with an initially lowered value increased (p = 0.02) and remained for 12 months (p<0.001).

**Conclusion:**

Revaccination with DT toxoid in children with GN not only does not cause undesirable changes in the system of immunocompetent cells but also has an immunomodulatory effect, which contributes to the favorable maintenance of the remission period of the disease.

## Introduction

Glomerulonephritis (GN) is a chronic kidney disease with impaired structure or function. With unresolved inflammation, kidney damage eventually progresses to chronic kidney disease (CKD) (1–3). Inflammation, a process essentially directed at detecting and controlling harmful pathogens, is a major pathogenic mechanism for both CKD and acute kidney injury. The kidneys contain many resident immune cells that play an important role in maintaining tissue homeostasis. Among them, there are dendritic cells (DC), macrophages, regulatory T-cells (Treg), cytotoxic lymphocytes (CD8+), and NK cells, which are in close contact with parenchymal cells (4, 5). Innate immune cells, especially resident DCs, play a corresponding role in damage and repair at the site of injury (6). Studies show that the death (lysis) of kidney cells is accompanied by the release of endogenous cytokines, chemokines associated with the emergence of molecular patterns (signals) of danger/damage (DAMP) as a result of oxidative stress. The release of these biologically active substances significantly contributes to the infiltration and activation of immune cells, which leads to the development of CKD (7–10). That is, after exposure to external (for example, microbial antigens) or internal factors, these cells produce inflammatory mediators that can initiate kidney disease and, at the same time, trigger a regulatory response aimed at curbing inflammation, restoring tissue damage and homeostasis.

There is ample evidence that functional impairments in monocytes, neutrophils, dendritic cells, and Th-lymphocyte maturation are directly related to the risk of increased infection rates during CKD. It is known that timely qualified treatment, preventive and rehabilitative measures started in early childhood can contribute to a favorable course and outcome of the disease. The role of vaccination in the prevention of infectious diseases in patients with kidney disease is undeniable and has been proven in numerous studies (11–16). A wide range of publications notes the safety of vaccine administration with the likely development of moderate general and local reactions in the post-vaccination period and indicates the possibility of the formation of specific antibodies to vaccine components; the relationship between the synthesis of post-vaccination antibodies and the duration of their preservation, taking into account the received immunosuppressive therapy; selection of the optimal timing of remission of the disease for the administration of live attenuated and killed vaccines; ways to improve the tactics of increasing the coverage of vaccination of such patients, etc. However, the mechanisms of immune reactions that occur during the administration of various vaccine preparations remain insufficiently studied. Their identification is especially important for justifying vaccination and clarifying the issue of incomplete effectiveness of immunobiological agents, intending to further improve the technologies for their production and the approach to vaccination of immunocompromised patients.

Anti-diphtheria and anti-tetanus toxoids with a reduced content of antigens in one vaccination dose are widely used in the practice of immunization for boosterization (revaccination) and are considered one of the safest and sufficiently immunogenic when used not only in healthy persons but in patients with deviations in health (17). In our opinion, at present during a pandemic, the circulation of the SARS-COV-2 virus should not cause concern on the part of medical personnel and serve as a medical counterindication from routine vaccination of children with chronic diseases against other infections based on the idea that in the post-vaccination period anti-infectious immunity decreases. In this work, we wanted to reveal the influence of the introduction of vaccine antigens on nonspecific factors of protection of vaccinated children in the post-vaccination period. So, this study aimed to investigate the effect of revaccination against diphtheria and tetanus on the proliferation and differentiation of immunocompetent cells, the formation of specific antibodies, and the course of the disease in children with glomerulonephritis.

## Materials

### Clinical Study Design

The main aim of the study was to analyze the effect of the vaccine against diphtheria and tetanus on the proliferation and differentiation of immunocompetent cells in children with glomerulonephritis (GN) in remission during revaccination with DT toxoid (Diphtheria-Tetanus toxoid with reduced antigenic content), as well as the ability to form specific antibodies (AT) and assess the clinical tolerance of vaccination in the early stages (1-30 days). Secondary outcomes included the assessment of the dynamics of the formation of adaptive immunity with the study of the subpopulation composition of T-lymphocytes and the levels of antitoxic antibodies (6-12 months) against diphtheria and tetanus, as well as characterizing the remission period of the underlying disease after vaccine administration.

The study included 45 children who were divided into vaccinated (25 children) and control (20 children) groups.

Stage IV (post-marketing), a non-randomized, controlled study of observers was carried out in two centers in Moscow (Russia): Children’s City Clinic No. 119 and in the Clinical Center for Immunoprophylaxis of Children’s Infections of the I.I.Mechnikov Research Institute of Vaccines and Sera. The selection of patients for the study group was carried out in the nephrological and polyclinic departments of the Research Institute of Pediatrics of the Russian Academy of Medical Sciences, the Research Institute of Pediatrics and Pediatric Surgery of the Ministry of Health of the Russian Federation, the nephro-urological center at the Children’s Clinical Hospital No. 13, the office of the district nephrologist of the children’s polyclinic No. 131. All children were registered with a nephrologist. After preliminary studies and a doctor’s examination, taking into account the indications and contraindications according to the instructions attached to the vaccine, as well as official recommendations for immunization in the Russian Federation, the patient was sent to the immunization room, where the nurse performed the vaccination in compliance with aseptic, antiseptic and drug administration rules. After vaccination, the patient was observed in a medical facility for 45 minutes to exclude the likelihood of developing immediate reactions to the vaccine. For 10 days, the doctor daily by phone asked the vaccinated about his health, if unusual phenomena occurred, an outpatient appointment, and consultation of the patient was carried out. Vaccinated and unvaccinated children were closely monitored for at least 1 year. All information about the vaccination, examination, and research data was recorded in the official, standard individual, medical documentation of the patient, where not only the attending nephrologist but also the healthcare managers knew about the study.

### Legal and Ethical Aspects of Research

Vaccination of patients with GN was carried out following the National Schedule of Preventive Vaccinations of the Russian Federation - a regulatory legal act establishing the timing and procedure for conducting preventive vaccinations for citizens (Federal Law of 17.09.1998 N 157-FZ); Article 20 - “Informed voluntary consent to medical intervention and refusal of medical intervention” (Federal Law No. 323-FZ dated 01.11.2011 on the basics of protecting the health of citizens in the Russian Federation (amended from 03.04.2017); Methodological guidelines MU 3.3.1.1123 -02 “Monitoring of post-vaccination complications and their prevention” approved by the Chief State Sanitary Doctor of the Russian Federation on May 26, 2002, paragraph 9.6.4. - Kidney diseases: children with chronic urinary tract infection, including chronic glomerulonephritis, pyelonephritis, vaccinated in remission with minimal changes in urine analysis, against the background of supportive antibiotic therapy.

The study protocol was approved by the local Ethics Committee of the I.I.Mechnikov Research Institute of Vaccines and Sera and the study was conducted under the Declaration of Helsinki, the International Council for Harmonization Guidelines for Good Clinical Practice, and Russian regulatory requirements. Written informed consent was obtained from the parents before the enrollment of their children in the study.

### Clinical Laboratory Examination

All children included in the study underwent a control examination to confirm the condition of complete clinical and laboratory remission of the underlying disease. The generally accepted set of diagnostic measures included a consultation with a nephrologist, 2-3 general urinalysis, a test according to Nechiporenko or Addis-Kakovsky, determination of the level of daily proteinuria.

Also, all patients underwent a study of the immune status: the number of lymphocytes and their subpopulations, the initial level of specific antibodies against diphtheria, and tetanus.

Revaccination of DT with toxoid was carried out at the FSBSI Mechnikov Research Institute of Vaccines and Serums.

### Vaccinated Children Follow-Up

For the vaccinated children, an in-depth clinical observation was carried out for 12 months with the participation of a pediatric nephrologist to assess the possible effect of the injected toxoid on the course of the underlying disease. The volume of laboratory tests was similar to that carried out before the administration of the vaccine preparation (general urinalysis, Nechiporenko test, or Addis-Kakovsky test, determination of the level of daily proteinuria). During the first month after vaccination, urine tests were done every 10 days, then quarterly during the year.

In the group of immunized children, all types of reactions that occur in response to the administration of DT toxoid were studied, including the reactions provided for in the instructions for use of the drug. At the same time, the general condition of the child was taken into account, as well as changes in organs and systems. In addition, the incidence of acute respiratory infections (ARI) in children in the first month of the post-vaccination period was also studied.

For serological control of the effectiveness of the revaccination performed 1-1.5 and 12 months after the administration of the vaccine preparation, the level of anti-diphtheria and anti-tetanus IgG antibodies was studied in all children. A control study of the immune status was carried out at the same time.

Controls (i.e., unvaccinated children) underwent the same comprehensive examination of the underlying disease at the same time. Since these children did not receive vaccination, respectively, the level of postvaccinal specific antibodies cannot change, and, in this regard, we did not consider it necessary to provide redundant (unnecessary) information in figures and tables. Blood sampling and vaccination were carried out in compliance with all the aseptic and antiseptic regulations using disposable instruments and sterile test tubes in a treatment room of the FSBSI Mechnikov Research Institute of Vaccines and Serums.

### The Structure of Diseases and Characteristics of Groups of Children

The clinical course of the post-vaccination period was studied in 45 children with glomerulonephritis (GN) aged 5 to 15 years. Of these, 25 children were revaccinated with DT toxoid and 20 were in the control group (not vaccinated). The age of children in the vaccination group was 10 (7-13) years, in the control group – 11.5 (9-14) years (U = 204, p = 0.29). In terms of gender distribution, the groups were also comparable with each other (χ^2^ = 0.01, p = 0.93): the proportion of girls in the vaccinated group was 24% (6 subjects), in the control group, it was 25% (5 subjects).

According to the nosological forms of the disease, children with GN were distributed as follows: 15 subjects in the study group and 11 subjects in the control group (60% and 55%, respectively, χ^2^ = 0.11, p = 0.73) had a nephrotic form of chronic GN, 4 subjects in each group (16% and 20%, respectively, χ^2^ = 0.12, p = 0.72) had a hematuric form of GN and 7 subjects in the study group and 5 in the control group (28% and 25%, respectively, χ^2^ = 0, 05, p = 0.82) suffered from acute GN.

Disease duration ranged from 2 to 7 years: 5 (5-6) years in the revaccinated group and 4 (3-6) years in the control group (U = 199, p = 0.24). At the same time, the duration of the remission period varied from 6 months to 4 years, being 2.3 (1.6-2.8) years in the study group and 2.6 (1.9-3.4) years in the control group (U = 197, p = 0.23). In 76% of cases (19 subjects) in the vaccinated group and 85% of cases (17 subjects) in the control group, the manifestations of GN were stopped only with the prescription of immunosuppressive drugs; the differences between the groups were not statistically significant (χ^2^ = 0.56, p = 0.45). It should be noted that in 10 children with the nephrotic form (4 in the control group and 6 in the study group, χ^2^ = 0.28, p = 0.59), the disease had a persistent recurrent nature, and remission was achieved only under conditions of combined therapy with glucocorticoids and cytostatics.

### The Nature of Comorbidities in Children

Analyzing information about the nature of comorbidities in children with GN, we noted a high percentage (40% – 18 subjects) of allergic diseases. Among the patients described by us, 10 subjects had atopic dermatitis and 8 had hay fever, which in 5 children manifested itself in the form of rhinoconjunctival syndrome, and in 3 – in the form of urticaria. Related diseases are presented in **Table 1**. The study groups are comparable in terms of the incidence of each of the diseases under consideration.

**Table 1:** The incidence of certain comorbidities in the study groups.

Complications			Study groups				Between groups^1^
	Total		Vaccinated		Control		
	Abs.	%	Abs.	%	Abs.	%	
Atopic dermatitis	10	22%	6	24%	4	20%	χ^2 =^ 0.10, p=0.75
Hay fever	8	18%	5	20%	3	15%	χ^2 =^ 0.19, p=0.66
Rhinoconjunctival syndrome	5	11%	3	12%	2	10%	χ^2 =^ 0.04, p=0.83
Urticaria	3	7%	2	8%	1	5%	p=1.00
Metabolic disorders	16	36%	8	32%	8	40%	χ^2 =^ 0.31, p=0.58
Hyperuraturia	9	20%	5	20%	4	20%	χ^2 =^ 0.01, p=1.00
Hyperoxaluria	7	16%	3	12%	4	20%	χ^2 =^ 0.54, p=0.46
Enuresis	3	7%	1	4%	2	10%	p=0.58
Frequent ARI	21	47%	12	48%	9	45%	χ^2 =^ 0.04, p=0.84
Diseases of the gastrointestinal tract	20	44%	13	52%	7	35%	χ^2 =^ 1.30, p=0.25

^1^ – Chi-Square test was applied (Fisher’s test in the presence of cells with frequencies less than 5%).

### Medical Support for Revaccination of Children

The prevalence of allergic pathology and the possible involvement of hypersensitivity reactions in the development or progression of GN determined the need for drug preparation for the administration of a vaccine preparation, its volume, and duration. For 5 days before and after vaccination, all children were prescribed one of the antihistamines (Fencarol, Claritin, and others) at an age-specific dosage. In addition, patients with allergic diseases were vaccinated against the background of a drug that stabilizes mast cell membranes: ketotifen (zaditen) 1 mg 2 times a day 3 weeks before and 5 weeks after revaccination. In the case of pollinosis, vaccinations were made outside the period of seasonal exacerbation.

### Vaccinal Status

For children with GN, revaccination was performed with a period of complete clinical and laboratory remission of at least 2 months. Patients already had a history of 2 to 4 doses of diphtheria, tetanus, and/or pertussis vaccine before being diagnosed with the disease. These patients did not receive routine revaccination against diphtheria and tetanus, as well as against other infections. At the time of examination, more than 5 years had passed in all patients from the last injection of diphtheria-tetanus toxoid and/or whooping cough. In most cases, this period was 6-7.5 years, in 2 people it was prolonged to 8.5 and 9 years, and in 1 child – up to 12 years 7 months. The difference between the control group and the study group was not statistically significant for this parameter: 6.5 (5.5-7.1) – in the control group, 6.6 (5.9-7.5) – in the study group (U = 222.5, p = 0.54).

It should be emphasized that a preliminary analysis of the initial levels of post-vaccination IgG antibodies did not reveal any dependence of the parameters of specific anti-diphtheria and anti-tetanus immunity on the data of the medical history or vaccine history.

In none of the previously vaccinated children with GN, prior vaccination was the cause of the development of the disease or its exacerbation (the minimum period from the moment of immunization to the onset of symptoms of the activity of the pathological process was 4.5 months).

### Vaccine

To immunize children, a national drug (i.e., a purified adsorbed diphtheria-tetanus toxoid with a reduced antigen level [DT toxoid]) produced by Biomed JSC named after I.I. Mechnikov (Russia) was used. A vaccination dose (0.5 mL) contains 5 flocculating units (FU) of diphtheria and 5 antitoxin-binding units (AU) of tetanus toxoid. Children were not revaccinated against pertussis, since combined vaccines with an acellular pertussis component have not been registered in our country. Moreover, a DTaP vaccine containing a whole-cell component is indicated only to children under 4 years old.

Children were revaccinated with DT toxoid (0.5 mL, i/m, into the shoulder deltoid) once.

### Exclusion Criteria

Children in the acute period of the disease or remission of the disease less than 2 months (to exclude the presence of changes in blood and urine tests); acute respiratory infections within last 2 The drug safety was assessed by individual examination and questioning of all the vaccinated patients, as well as the recording of local and systemic reactions to DT toxoid within 14 days and in 1 month after the vaccination.

### Leukocyte’s Count

Peripheral blood mononuclear leukocytes (PBMLs) were isolated from whole blood in a ficoll-urographin density gradient (10^6^ cells per 1 ml of RPMI-1640 medium (PanEko, Russia)).

### Subpopulation Structure of Lymphocytes

Subpopulation structure of peripheral blood lymphocytes was investigated *in vitro* by flow cytometry with Cytomix FC-500 (Beckman Coulter, USA) using monoclonal antibodies (mAbs) to CD3-FITC/CD16/56-PE, CD45-FITC/CD3-ECD/CD20-PE, CD45-FITC/CD3-PE/CD4-PC5, CD45-FITC/CD3-PE/CD8-PC5 (Immunotech, France**).** As a comparison, we used pediatric age-specific standards obtained in the clinical and immunological laboratory of the Research Institute of Children Oncology and Hematology Federal State Budgetary Institution Blokhin National Medical Research Center of Oncology of the Ministry of Healthcare of the Russian Federation and the Federal State Budgetary Institution State Scientific Center Institute of Immunology of the FMBA of the Russian Federation.

### Determination of the Level of IgG Antibodies to Diphtheria and Tetanus

The serum level of specific antibodies to diphtheria and tetanus was investigated by enzyme-linked immunosorbent assay (ELISA) using commercial kits designed for assay of IgG antibodies (Euroimmun AG, Germany) (anti-diphtheria toxoid (*Anti-Diphtheria Toxoid* ELISA, IgG) and anti-tetanus toxoid (*Anti-Tetanus toxoid* ELISA, IgG)). According to the manufacturer’s instructions, reference values of anti-diphtheria immunity are as follows: <0.1 IU/mL – negative; 0.1-1.0 – short-term protection, boosting is required; >1.0 – positive. As for anti-tetanus immunity, reference values are as follows: <0.01 IU/mL – negative; 0.01-0.1 – doubtful; 0.1-0.5 – short-term protection, boosting is required; >0.5 – positive.

### Statistical Analysis

The data check for compliance with the normal distribution law (Shapiro-Wilks test) showed that the distribution of most of the investigated parameters was different from the normal one. Descriptive statistics of absolute and percentage of certain lymphocyte types is presented by the median and interquartile range (Me(Q1-Q3)). Descriptive statistics of the level of IgG antibodies is presented by the geometric mean and 95% confidence interval (GMC [95% CI]).

The analysis of repeated measurements of the level of IgG antibodies at control points of the study was carried out with Friedman’s nonparametric one-way analysis of variance for linked samples. Multiple post-hoc comparisons of the values with the pre-revaccination level at the control points were performed with the Demsar method. The Mann-Whitney criterion (U) was used to compare the two study groups on a quantitative basis. The median value was compared with the normal range with the Wilcoxon signed-rank test. Comparison of independent samples by qualitative nominal parameters was carried out during the analysis of contingency tables using the Pearson Chi-square test (Fisher’s exact test in the presence of cells with frequencies less than 5%).

To analyze the change in the number of individual types of lymphocytes depending on the period and study group (control and vaccinated), a linear mixed-effects model (LMEM) was used. The prerequisites for use were checked by the Levigne test (homoscedasticity of residues) and the Shapiro-Wilks test (normality of distribution of standardized residues). Also, a linear model of mixed-effects was built by adding a third influencing factor: the initial level of the analyzed parameter. Post hoc tests were performed using Tukey’s test within the same study group, depending on time after vaccination and within the same checkpoint between study groups. Comparison of the number of subpopulations of lymphocytes in the group of subjects, vaccinated 12 months after immunization, between patients with different baseline levels of the analyzed parameter was carried out by the Mann-Whitney test (if two baseline levels were compared) and the Kruskal-Wallis test (if three baseline levels were compared).

Differences were considered statistically significant at p ≤ 0.05. All the calculations were performed in the free R statistical environment (v.3.6, GNU GPL2 license).

## Results

### Post-Vaccination Clinical Course

In the majority of children (92%) with GN, the early and late post-vaccination periods were uneventful. Only in 2 cases (8%) we observed mild general reactions in the form of a rise in body temperature to 37.3^°^C in the first three days after vaccination, which did not require the prescription of any drug therapy. Local post-vaccination reactions were not observed in this group of patients.

ARI was registered in 2 children (8%) during the first month after vaccination. In the first case, the disease began on the 6-7th day after the injection of DT toxoid, proceeded without complications, and resolved within a week against the background of symptomatic therapy. In another child, the first symptoms of ARI were noted on the 13th day of vaccination. The disease was accompanied by fever up to 38.4^°^C, intoxication, and catarrhal symptoms. After 2 days, acute bronchitis was diagnosed, and, in this regard, in addition to symptomatic, antibiotic therapy was prescribed (cefixime at an age dosage of 7 days). By the 10th day from the onset of the disease, all its manifestations resolved.

When observing revaccinated children for a year or more, there was no increase in the frequency or severity of the course of intercurrent diseases in comparison with the previous year.

In the early post-vaccination period, we did not notice any exacerbation of GN or concomitant disease in any of the patients.

### Post-Vaccination Antibody Level

**Figure 1:**
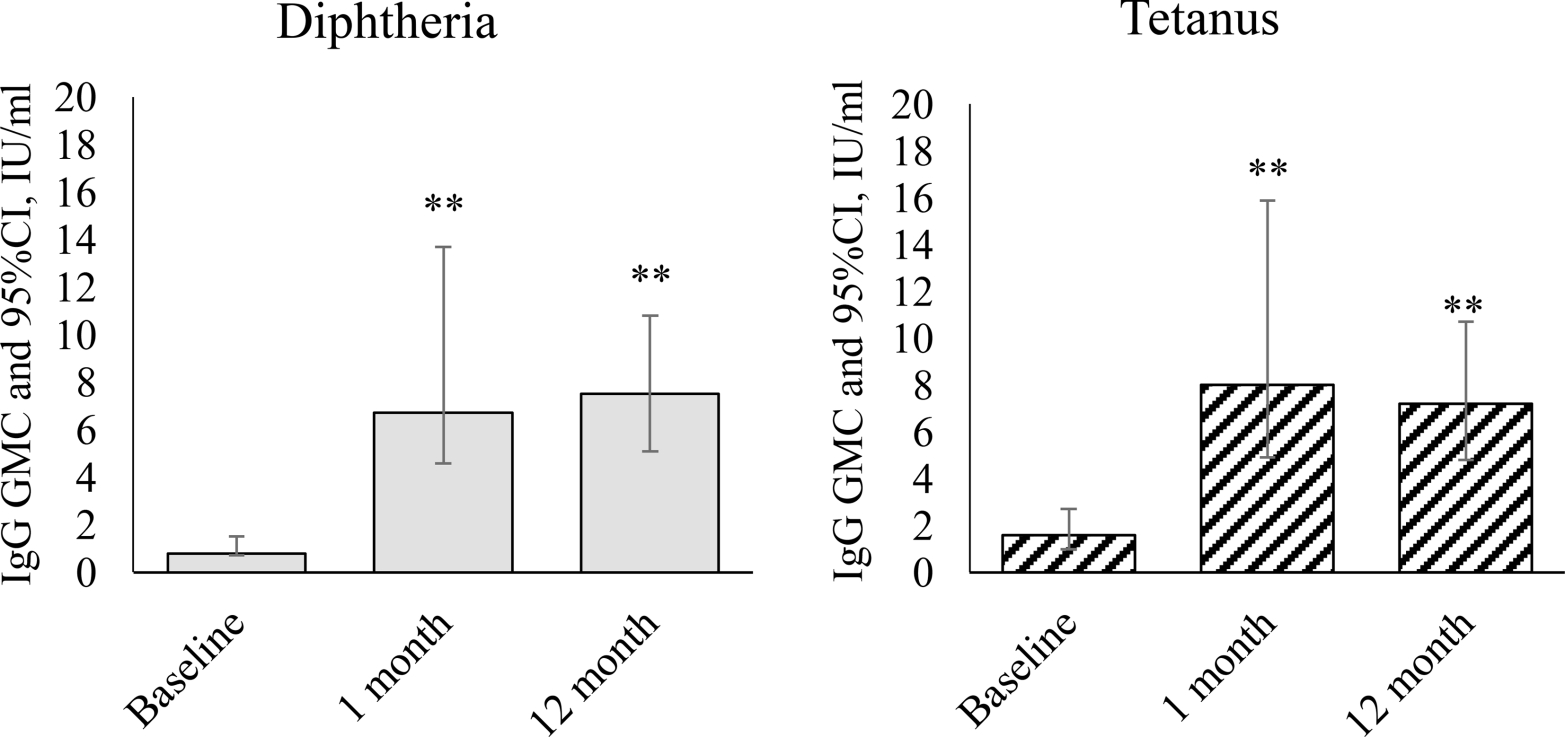
Dynamics of IgG antibodies against diphtheria and tetanus during revaccination with DT toxoid (n = 25). Geometric mean concentrations and their 95% confidence interval (GMC [95% CI]). ** – differences from the pre-revaccination value at a level of p < 0.001 (we used the post-hoc Demsar test with a statistically significant Friedman omnibus test).

The results of studies of specific immunity in children with GN revaccinated with DT toxoid are presented in **Figure 1**.

After vaccination, we observed a statistically significant increase in the level of IgG antibodies against diphtheria ( $\chi_{\text{r}}^{2}$ = 31.1, p<0.001) and against tetanus ( $\chi_{\text{r}}^{2}$ =19.4, p<0.001). Before revaccination, the level of antibodies against diphtheria was 0.8 [0.7-1.5] IU/mL, against tetanus – 1.6 [1.0-2.7] IU/mL. A month after revaccination, the level of antibodies against diphtheria increased relative to the initial level to 6.7 [4.6-13.7] IU/mL (p<0.001), against tetanus to 8 [4.9-15.9] IU/mL (p<0.001). 12 months after revaccination, the level of antibodies against both diphtheria and tetanus remains at a high level: 7.5 [5.1-10.8] IU/mL – against diphtheria (p<0.001 relative to the initial level), 7.2 [4.8-10.7] IU/mL – against tetanus (p<0.001).

### Subpopulation Structure of T-Lymphocytes During the Revaccination

The study of the initial parameters of the average values of leukocytes, lymphocytes and the relative and absolute values of subpopulations of lymphocytes did not reveal any significant difference in children with GN from normal values (**Table 2**).

**Table 2:** Comparative characteristics of peripheral blood lymphocytes subpopulation structure in children with GN and healthy ones aged 7-15 years.

Parameters	Healthy children (normal range)	Patients with GN^1^			p^3^ (including the normal range)	
		Vaccine (n=25)	Control (n=20)	p^2^	Vaccine	Control
Leukocytes	6000 (5024-6970)	6773 (5148-7635)	6627 (5724-7090)	0.95	0.21	0.07
Lymphocytes, %	39.5 (35-43)	38 (36-41)	39 (36-43)	0.80	0.14	0.13
Lymphocytes, abs.	3500 (2350-4620)	2827 (2351-4160)	2815 (2362-4057)	0.63	0.53	0.10
CD45/СD3+, % %	71 (65-78)	76 (68-84)	74 (66-80)	0.22	0.09	0.18
CD45/СD3+, % abs.	1700 (1312-2028)	1810 (1549-2190)	1735 (1501-1961	0.16	0.07	0.21
CD45/CD3/СD4+, %	37 (32-41)	40 (33-44)	40.5 (33-45)	0.74	0.08	0.15
CD45/CD3/СD4+, abs	900 (750-1100)	1038 (817-1247)	1074 (853-1247)	0.98	0.10	0.07
(CD45/CD3/СD8+), %	31 (25-34)	29 (21-36)	29 (20-34)	0.12	0.07	0.18
(CD45/CD3/СD8+), abs	750 (601-854)	700 (604-789)	706 (538-774)	0.65	0.09	0.10
CD16/56+, %	12.5 (7-18)	8.5 (5-14)	10 (7-14)	0.98	0.10	0.06
CD16/56+, abs	250 (210-316)	242 (184-274)	222 (189-277)	0.73	0.25	0.06
CD45/CD20+, %	12.5 (8-16)	10 (6-14)	10 (8-14)	0.85	0.09	0.12
CD45/CD20+, abs	400 (325-494)	378 (318-449)	381 (322-426)	0.83	0.17	0.41
CD4/CD8 IRI	1.25 (0.8-1.7)	1.1 (0.9-1.5)	1.1 (0.9-1.3)	0.84	0.26	0.08

^1^medians and interquartile range: Me (Q1-Q3).

^2^patient groups were compared with the Mann-Whitney U-test.

^3^comparison with the normal range was carried out with the Wilcoxon signed rank test.

During the follow-up examination of patients in the control unvaccinated group and vaccinated patients, carried out 1 and 12 months after the start of the study, results were obtained that were almost similar to the initial data. In the study groups, no dynamics of parameters were revealed. The control group and the vaccinated group also did not differ between themselves (**Table 3**).

**Table 3:** Dynamics of the subpopulation structure of peripheral blood lymphocytes in children with GN after revaccination with DT toxoid (n = 25) and in the control group (n = 20).

Parameters	Group	The values of the parameter^1^ at control points Groups: revaccination (Rev) n=25, control (CG) n=20		
		Before Rev	After 1 month	After 12 months
Leukocytes	Rev	6773 (5148-7635)	6378 (5185-7170)	6488 (5534-7209)
	CG	6627 (5724-7090)	6741 (5865-7475)	6357 (5632-7303)
	LMEM^2^	Time: F=1.9, p=0.15, Rev: F=1,2, p=0.27		
		Time*vaccination: F=0.5, p=0.63		
Lymphocytes, %	Rev	38 (36-41)	38 (34-42)	37 (35-44)
	CG	39 (36-43)	38 (36-43)	39 (31-41)
	LMEM	Time: F=0.4, p=0.70 Vaccination: F=0.3, p=0.58		
		Time*vaccination: F=1.6, p=0.21		
Lymphocytes, abs	Rev	2827 (2351-4160)	2675 (2247-4379)	2705 (2278-3264)
	CG	2815 (2362-4057)	2751(2169-3915)	2801(2076-3484)
	LMEM	Time: F=1.5, p=0.24 Vaccination: F=0.6, p=0.43		
		Time*vaccination: F=0.4, p=0.69		
Т-lymphocytes (CD45/СD3+), %	Rev	76 (68-84)	76(71-85)	74(68-83)
	CG	74 (66-80)	76(73-78)	75(70-78)
	LMEM	Time: F=1.2, p=0.32 Vaccination: F=1.1, p=0.30		
		Time*vaccination: F=0.1, p=0.90		
Т-lymphocytes (CD45/СD3+), abs.	Rev	1810 (1549-2190)	1785(1346-2321)	1863(1622-1965)
	CG	1735 (1501-1961)	1765(1697-1951)	1788(1614-1909)
	LMEM	Time: F=0.5, p=0.62 Vaccination: F=0.1, p=0.71		
		Time*vaccination: F=1.5, p=0.24		
T-helpers (CD45/CD3/СD4+), %	Rev	40 (33-44)	41(34-42)	40(35-41)
	CG	40.5 (33-44)	40(33-51)	41(36-54)
	LMEM	Time: F=2.4, p=0.10 Vaccination: F=0.8, p=0.38		
		Time*vaccination: F=1.8, p=0.15		
T-helpers (CD45/CD3/СD4+), abs	Rev	1038 (817-1247)	1139(1023-1352)	1031(900-1159)
	CG	1074 (853-1247)	975(855-1145)	1052(896-1248)
	LMEM	Time: F=1.5, p=0.23 Vaccination: F=0.6, p=0.58		
		Time*vaccination: F=2.2, p=0.12		
Cytotoxic Т-lymphocytes, (CD45/CD3/СD8+), %	Rev	29 (21-36)	28(25-31)	26(25-30)
	CG	29 (20-34)	29(21-34)	28(21-34)
	LMEM	Time: F=0.2, p=0.66 Vaccination: F=2.0, p=0.14		
		Time*vaccination: F=0.3, p=0.74		
Cytotoxic Т-lymphocytes, (CD45/CD3/СD8+), abs	Rev	700 (604-789)	679(628-879)	653(510-819)
	CG	706 (538-774)	723(616-901)	705(619-859)
	LMEM	Time: F=0.1, p=0.78 Vaccination: F=1.6, p=0.21		
		Time*vaccination: F=0.6, p=0.57		
Natural killer cells, NK cells (CD16/56+), %	Rev	8.5 (5-14)	8(7-9)	7(6-12)
	CG	10 (7-14)	8(3-16)	9(5-12)
	LMEM	Time: F=0.1, p=0.93 Vaccination: F=2.8, p=0.06		
		Time*vaccination: F=2.7, p=0.06		
Natural killer cells, NK cells (CD16/56+), abs	Rev	242(184-274)	228(185-275)	246(188-278)
	CG	222(189-277)	233(192-255)	220(204-251)
	LMEM	Time: F=0.64, p=0.43 Vaccination: F=0.2, p=0.97		
		Time*vaccination: F=0.22, p=0.80		
B-cells (CD45/CD20+), %	Rev	10 (6-14)	9(7-12)	8(7-12)
	CG	10 (8-14)	7(7-9)	9(7-11)
	LMEM	Time: F=0.7, p=0.39 Vaccination: F=2.7, p=0.07		
		Time*vaccination: F=0.8, p=0.43		
B-cells (CD45/CD20+), abs	Rev	378(318-449)	373(297-468)	356(296-429)
	CG	381(322-426)	365(318-471)	387(275-463)
	LMEM	Time: F=0.2, p=0.9 Vaccination: F=0.2, p=0.85		
		Time*vaccination: F=0.6, p=0.54		
CD4/CD8 IRI	Rev	1.1(0.9-1.5)	1.1(1-1.3)	1.4(0.9-1.7)
	CG	1.1(0.9-1.3)	1.4(1-1.5)	1.4(0.8-1.6)
	LMEM	Time: F=0.1, p=0.94 Vaccination: F=1.8, p=0.17		
		Time*vaccination: F=1.2, p=0.32		

^1^medians and interquartile range: Me (Q1-Q3).

^2^a linear mixed-effects model was used, where the period (control point) and the group are fixed factors and individual patients are random ones.

That is, the assessment of the average relative and absolute values of lymphocyte subpopulations in children with GN did not allow us to identify in their changes in the content of immunocompetent cells characteristic of this pathology before and in the dynamics of the vaccination process. Probably, the absence of such changes was due to the leveling of differences in the subpopulation composition of peripheral blood lymphocytes in the initially heterogeneous groups when calculating the mean values.

To avoid possible errors, we analyzed the frequency of occurrence of normal and altered values of lymphocyte subpopulations in children with kidney diseases, for which we divided each of the examined groups into 3 subgroups: with normal, increased, and decreased values of the content of lymphocyte subpopulations. The results of studying the effect of DT toxoid on the ratio in these subgroups of the number of children with normal and altered content of immunocompetent cells are presented in **Table 4**.

**Table 4:** Dynamics of the subpopulation structure of peripheral blood lymphocytes in children with GN after revaccination with DT toxoid and the control group.

	Baseline^1^	Group	N	Parameter value^2^ in control points			p – between control points
				Before R	After 1 month	After 12 months	
CD45/СD3+, %	Limit	Rev	14	67(65-70)	71(66-73)	70(66-74)	p^1-0 =^ 0.25
							p^12-0 =^ 0.59
		CG	8	68(66-71)	71(68-74)	68(66-71)	p^1-0 =^ 0.28
							p^12-0 =^ 0.91
	p between groups			p=1.00	p=0.45	p=0.28	–
	Above normal	Rev	11	83(81-86)	78(76-80)	75(69-79)	p^1-0 =^ 0.04
							p^12-0^<0.001
		CG	10	84(82-87)	85(80-87)	83(80-86)	p^1-0 =^ 0.96
							p^12-0 =^ 0.49
	p between groups			p=0.28	p<0.001	p<0.001	–
	LMEM^3^			Time: F=2.1, p=0.13 Vaccine: F=2.2, p=0.15			
				Time*vaccine*baseline: F=3.1, p=0.005			
CD45/CD3/СD4+,	Limit	Rev	14	37(34-40)	38(36-41)	38(36-42)	p^1-0 =^ 0.42
							p^12-0 =^ 0.54
		CG	12	37(32-39)	37(35-40)	35(33-39)	p^1-0 =^ 0.55
							p^12-0 =^ 0.96
	p between groups			p=0.54	p=0.58	p=0.29	–
	Above normal	Rev	11	50(48-52)	46(39-48)	40(38-43)	p^1-0 =^ 0.003
							p^12-0^<0.001
		CG	8	50(47-52)	49(45-50)	50(48-55)	p^1-0 =^ 0.55
							p^12-0 =^ 0.55
	p between groups			p=0.63	p=0.04	p<0.001	–
	LMEM			Time: F=0.4, p=0.53 Vaccine: F=2.5, p=0.09,			
				Time*vaccine*baseline: F=11.9, p<0.001			
CD45/CD3/СD8+, %	Below normal	Rev	8	19(18-21)	23(17-30)	26(21-32)	p^1-0^<0.001
							p^12-0^<0.001
		CG	6	20(19-21)	21(17-24)	22(20-25)	p^1-0 =^ 0.87
							p^12-0 =^ 0.67
	p between groups			p=0.87	p<0.001	p=0.005	–
	Limit	Rev	10	28(27-30)	28(25-31)	27(26-30)	p^1-0 =^ 0.71
							p^12-0 =^ 0.40
		CG	8	29(28-30)	29(27-32)	28(26-30)	p^1-0 =^ 0.66
							p^12-0 =^ 0.42
	p between groups			p=0.66	p=0.87	p=0.66	–
	Above normal	Rev	7	35(34-37)	30(28-34)	28(22-34)	p^1-0^<0.001
							p^12-0^<0.001
		CG	6	39(35-40)	36(35-38)	36(34-37)	p^1-0 =^ 0.34
							p^12-0 =^ 0.33
	p between groups			p=0.34	p=0.09	p<0.001	
	LMEM			Time: F=2.0, p=0.16 Vaccine: F=1.6, p=0.22,			
				Time*vaccine*baseline: F=5.3, p=0.001			
CD45/CD20+, %	Below normal	Rev	5	5(4-6)	6(4-8)	8(6-11)	p^1-0 =^ 0.02
							p^12-0^<0.001
		CG	5	6(3-7)	5(4-6)	6(4-7)	p^1-0 =^ 0.91
							p^12-0 =^ 0.82
	p between groups			p=0.83	p=0.30	p=0.002	–
	Limit	Rev	20	11(9-15)	9(8-12)	10(9-13)	p^1-0 =^ 0.31
							p^12-0 =^ 0.30
		CG	15	12(9-14)	8(7-11)	10(7-12)	p^1-0 =^ 0.82
							p^12-0 =^ 0.30
	p between groups			p=0.91	p=0.87	p=0.91	–
	LMEM			Time: F=0.4, p=0.65 Vaccine: F=2.2, p=0.09,			
				Time*vaccine*baseline: F=5.3, p=0.007			

^1^If the number of patients with a certain baseline level was less than 5, then this level was excluded from consideration.

^2^Geometric mean concentration and 95% confidence interval of GMC are given [95% CI].

^3^A linear mixed-effects model was used, where time (control points), group, baseline parameter were fixed factors and individual patients were random ones. Post hoc comparisons (between groups at control points and between control points for each group) were carried out using Tukey’s test, where p^1-0^ is a comparison of the initial value of the parameter and one month after revaccination; p^12-0^ – comparison of the initial value of the parameter and 12 months after revaccination.

**Figure 2:**
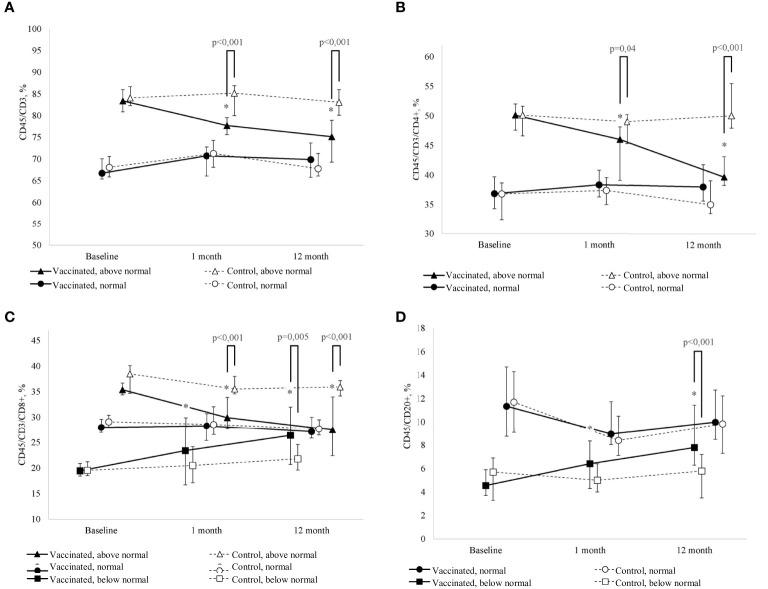
**(A)** Change in the percentage of T-lymphocytes (CD45/CD3+) of peripheral blood in dynamics in children with GN in the study group (after revaccination with DT toxoid – n = 25) and the control group (n = 20); the median and interquartile range are given: Me (Q1-Q3). * – statistically significantly different from the pre-vaccination value (p < 0.05). Tukey's test was used in a linear mixed effects model. **(B)** Change in the percentage of T-helpers (CD45/CD3/СD4+), of peripheral blood in children with GN in the study group (after revaccination with DT toxoid –n = 25) and the control group (n = 20); the median and interquartile range are given: Me (Q1-Q3). *p ≤ 0.05 – compared to the pre-vaccination level (p ≤ 0.05). Tukey's test was used in a linear mixed effects model. **(C)** Change in the percentage of cytotoxic T-lymphocytes (CD45/CD3/CD8+) of peripheral blood in dynamics in children with GN in the study group (after revaccination with DT toxoid –n = 25) and the control group (n = 20); the median and interquartile range are given: Me (Q1-Q3). #* – statistically significantly different from the pre-vaccination value (p ≤ 0.05). Tukey's test was used in a linear mixed effects model. **(D)** Change in the percentage of B-cells (CD45/CD20+) of peripheral blood in dynamics in children with GN in the study group (after revaccination with DT toxoid –n=25) and the control group (n=20); the median and interquartile are given: Me (Q1-Q3). * – statistically significantly different from the pre-vaccination value (p ≤ 0.05). Tukey’s test was used in a linear mixed effects model.

As a result of the study, it was revealed that the percentage of T-lymphocytes on average for the period under consideration in the revaccinated group and the control group did not differ (F=2.2, p=0.15), while no statistically significant dynamics was found in the study groups either (F=2.1, p=0.13) (**Table 4**, **Figure 2A**). However, when considering the groups taking into account the initial level of T-lymphocytes, in the study groups there is a statistically significant multidirectional change in their content over time (F=3.1, p=0.005). In particular, it was revealed that the initially increased percentage of T-lymphocytes as a result of revaccination with DT toxoid showed a statistically significant decrease: from 83 (81-86)% to 78 (76-80)% a month after revaccination (p=0.04) and up to 75 (69-79)% after 12 months (p<0.001). In the control group, such a decrease was not observed. Therefore, in the group of revaccinated patients and the control group, there are statistically significant differences in the study periods (1 and 12 months after the start of the observation, p<0.001).

Comparative analysis of T-lymphocytes from patients with initially elevated and normal levels 12 months after revaccination did not reveal significant differences (U=61, p=0.37). A decrease in the relative level of the content of T-lymphocytes is noted only at its initially increased levels; in the group of patients with an initially normal content of T-cells, no statistically significant differences were found both in the group of revaccinated subjects and in the control group.

A similar pattern is observed for T-helpers (**Table 4**, **Figure 2B**). When considering the groups, taking into account the initial level, there is a statistically significant multidirectional dynamic change in the percentage of T-helpers in the study groups (F=11.9, p<0.001). Already one month after revaccination, there is a significant (p=0.003) decrease in the initially increased content of T-helpers in the group of revaccinated persons, 12 months after revaccination, the decrease becomes even more pronounced: from 50 (48-52) % initially to 40 (38-43) % (p<0.001). In revaccinated patients with an initially elevated level of T-helpers compared with the control group after 1 month and 12 months, statistically significant differences were noted (p=0.04 and p<0.001, respectively). It should be noted that 12 months after revaccination, in patients with an initially increased content of the relative number of T-helpers, this parameter becomes comparable to the group of patients with an initially normal level (U=53, p=0.20). With the initially normal level of T-helpers, no statistically significant changes in this parameter were revealed as a result of revaccination.

Analysis of the percentage of cytotoxic T-lymphocytes revealed that despite the absence of differences in the mean values of the parameter depending on the revaccination (F=1.6, p=0.22) and the study period (F=2.0, p=0.16), there are statistically significant differences in the dynamics of the parameter depending on the initial level (F=5.3, p=0.001) (**Table 4**, **Figure 2C**). A month after revaccination, patients with an initially increased percentage of cytotoxic T-lymphocytes show a statistically significant decrease in this parameter (p<0.001), while patients with an initially low percentage of cytotoxic T-lymphocytes, on the contrary, have a statistically significant increase (p<0.001). As a result, the percentage of cytotoxic T-lymphocytes in the blood in patients with an initial value of this parameter lying outside the normative range, 12 months after revaccination, became comparable to the value of this parameter in the group of patients with an initially normal percentage of cytotoxic T-lymphocytes in the blood (H = 0.54, p=0.76).

The previously identified patterns are also observed in the change in the dynamics of the percentage of B-cells: there is a multidirectional change depending on the initial level of the parameter and vaccination (F=5.3, p=0.007) (**Table 4**, **Figure 2D**). One month after revaccination, the relative level of B-cells in the blood of patients with initially lowered levels increases (p=0.02). This growth persists for 12 months after revaccination (p<0.001). As a result, the level of B-cells in vaccinated patients with their initially lowered level reaches values higher (p=0.002) than the control and is comparable to the initially normal percentage of B-cells (U=30, p=0.09).

## Discussion

The post-vaccination period was assessed by the frequency of development of general and local reactions or complications, the addition of intercurrent diseases, and the severity of their course. In children with GN, after revaccination with DT toxoid, only isolated mild, short-term temperature reactions developed, which can also occur in practically healthy children, as provided in the instructions for use of the drug. Respiratory infections during the month were also rare and were detected only in 2 (8%) revaccinated children. It should be noted that one of them had frequent ARIs even before the drug was administered, therefore, in this case, we cannot testify about a more severe course of a respiratory infection. During the year, no increase in the incidence of ARIs was found in children with GN in comparison with the previous year before revaccination with DT.

The most important was the assessment of the development of late reactions within 1 year after the administration of the DT. In the case of vaccination of children with such a severe pathology as GN, the safety criterion for the administration of diphtheria-tetanus toxoid may be the absence of a relapse of the disease in the post-vaccination period. Before revaccination, all children underwent a control examination to confirm the condition of complete clinical and laboratory remission of GN. The generally accepted set of diagnostic measures included a consultation with a nephrologist, 2-3 general urinalysis, a test according to Nechiporenko or Addis-Kakovsky, determination of the level of daily proteinuria. A similar examination was carried out in the post-vaccination period to assess the possible negative effect of the injected toxoid on the course of the underlying disease. During the first month after vaccination, urine tests were done every 10 days, then quarterly during the year. It should be especially noted that during the dynamic study of urine analyzes for 1 month, as well as for one year, we did not reveal pathological changes in them.

When observing patients with GN, vaccinated with DT toxoid, the development of an exacerbation of the disease after vaccination was not noted, as well as changes in the clinical course of certain concomitant pathologies in the study groups. Of interest are children with GN and those with allergic diseases (40% - 18 people), which significantly exceeds this indicator on average in the population. These data are consistent with the hypothesis that allergic reactions play a role in the pathogenesis of GBV and maybe the trigger or even cause of the development of the disease. We have not identified in the post-vaccination period an exacerbation of allergic pathology or the occurrence of unusual reactions among vaccinated patients. It can be assumed that the appointment of antiallergic drugs during revaccination with anti-diphtheria and anti-tetanus toxoids for children with GN and concomitant allergic pathology is favorable in the clinical course of the post-vaccination period, as well as during immunization of patients with only an allergic disease (11, 15, 18).

Even though children with GN belong to the immunocompromised group, the immunosuppressive therapy carried out is not accompanied by the complete extinction of specific antibodies in the case of vaccination before the development of the disease. By the time of the examination of our patients, an average of 6-7.5 years had passed since the last injection of diphtheria-tetanus toxoid and/or whooping cough. The study of the initial levels of IgG antibodies to diphtheria revealed them at low values of 0.8 [0.7-1.5] IU/mL, providing short-term protection and indicating the need for boosting, while the values for tetanus were 1.6 [1.0 -2.7] IU/mL and they remained in protective concentrations. Long-term persistence of post-vaccination anti-diphtheria and anti-tetanus antibodies within protective values in children with chronic diseases and immunocompromised patients was also observed by other authors (19–21). They showed that in children with nephrotic syndrome who had previously received primary immunization, after >5 years since the last vaccination, seroprotection rates for diphtheria, tetanus, pertussis, and measles were 86.8%, 93.4%, 31.6%, and 77.6% respectively. At the same time, high titers of seroprotection (1.0 IU/mL) for diphtheria were observed in 23.8%, and for tetanus – in 69.3% of patients. In children with steroid-resistant nephrotic syndrome, the antibody values were lower than in patients with steroid-sensitive nephrotic syndrome (22). This indicates that this cohort of patients requires the administration of additional doses of vaccines to increase the level of anti-infectious protection against vaccine-preventable diseases.

The revaccination we carried out with DT toxoid in children with GN was accompanied after 1 month by a significant (8 times) increase in IgG antibodies against diphtheria and tetanus (5 times) with their subsequent preservation at a high level (7.5 [5.1-10, 8] IU/mL and 7.2 [4.8-10.7] IU/mL, respectively) 12 months after revaccination. At the same time, no differences in the levels of the investigated specific antitoxic antibodies were revealed by the indicated date. From this it follows that the administration of a booster dose of the vaccine in patients with GN with a remission period of more than 2 months leads to the synthesis of antibodies against diphtheria and tetanus in rather high values, allowing to protect against these infections.

It is known that when a vaccine is administered to healthy people, along with the formation of a specific immune response, non-specific shifts in the system of immunocompetent cells may appear, which are manifested in a change in the number and functional activity of various subpopulations of lymphocytes (16, 18, 23–25). In children with kidney diseases, various variations of the quantitative values of lymphocyte subpopulations have been described (26–28), which, on the one hand, can be aggravated during the vaccination process, and on the other hand, cause an inadequate immune response to vaccine antigens. Based on the foregoing, we considered it expedient to study the pre-vaccination values of lymphocyte subpopulations in children with GN and the dynamics of their changes after the administration of DT toxoid. The study of baseline parameters of lymphocyte subpopulations is especially important in children with GN since most of them received therapy with immunosuppressive drugs. However, we were unable to identify any changes in the relative and absolute values of lymphocyte subpopulations characteristic of these diseases in the examined children. Perhaps this is because the study of the immune status in patients with GN was carried out during the period of remission of the pathological process. In addition, the absence of such changes can be explained by the fact that deviations from the norm in this contingent of children are multidirectional and, when calculating the mean values, they can level each other out.

Of course, interesting data were obtained when analyzing the dynamic changes in the relative values of lymphocyte subpopulations in subgroups of children with an initially impaired content of these cells. We not only did not reveal undesirable shifts in the system of immunocompetent cells in children with GN after administration of DT toxoid but also demonstrated a possible immunomodulatory effect of DT toxoid, which was expressed in the normalization of initially increased or decreased values of subpopulations of peripheral blood lymphocytes in the post-vaccination period.

The obtained results of the study are also relevant at the current pandemic stage, and it is fair to emphasize the position of WHO that the cessation of vaccination, even for a short time, will lead to an increase in the number of susceptible persons and an increase in the likelihood of outbreaks of vaccine-preventable infections. In this regard, measures should be taken to maintain a high level of population immunity; determine the number of children who have not received the correct doses of vaccines; develop an action plan for catch-up immunization, taking into account the specific local conditions. That is, it is extremely important to continue carrying out full-fledged vaccine prophylaxis in children and especially those suffering from chronic diseases not only with routine immunization but also in the event of adverse epidemic situations (29). After all, outbreaks of vaccine-preventable infections will lead to severe cases, which will increase the pressure on the healthcare system, which is already under great stress associated with the response to the COVID pandemic. That is, patients with GN in the period of remission should receive vaccination against diphtheria and tetanus since vaccination is accompanied by the restoration of functional changes in certain immunocompetent cells, which is important in the individual’s resistance when meeting infectious pathogens.

## Conclusion

Thus, based on the data obtained, we can conclude that in children with glomerulonephritis in the period of remission there are no significant impairments of the functional activity of the immune system: the ability to form specific immunity has not been lost; post-vaccination antibodies are produced and maintained at a sufficient level for a long time. Revaccination with DT toxoid in children with GN not only does not cause undesirable changes in the system of immunocompetent cells but also has an immunomodulatory effect, which may contribute to the favorable maintenance of the remission period of the disease.

## Data Availability Statement

The original contributions presented in the study are included in the article/supplementary material. Further inquiries can be directed to the corresponding author.

## Ethics Statement

The protocol was approved by the local Ethics Committee of the FSBSI Mechnikov Research Institute of Vaccines and Serums and the study was conducted following the Declaration of Helsinki, the International Council for Harmonization Guidelines for Good Clinical Practice, and Russian regulatory requirements. Written informed consent was obtained from parents before enrollment of their children in the study.

## Author Contributions

MK – the head of the project. NA- Methodology, Resources. AK - Writing - Review & Editing. OM - Data Curation. KM - Writing - Review & Editing. VP – Investigation. AV– Statistic Procedures, Formal analysis. All authors contributed to the article and approved the submitted version.

## Conflict of Interest

The authors declare that the research was conducted in the absence of any commercial or financial relationships that could be construed as a potential conflict of interest.

## Publisher’s Note

All claims expressed in this article are solely those of the authors and do not necessarily represent those of their affiliated organizations, or those of the publisher, the editors and the reviewers. Any product that may be evaluated in this article, or claim that may be made by its manufacturer, is not guaranteed or endorsed by the publisher.
